# Supplementary material for: Effects of dexmedetomidine as a perineural adjuvant for femoral nerve block: A systematic review and meta-analysis
Source: PLoS One. 2020 Oct 19;15(10):e0240561. doi: 10.1371/journal.pone.0240561 (PMC7571703; doi:10.1371/journal.pone.0240561)
Supplement: S1 File — (DOCX) [file pone.0240561.s002.docx]

# **S1 File. Search strategy**

**PubMed**

#1. Search “Dexmedetomidine” [Mesh]

#2. Search “Dexmedetomidine” [Title/Abstract] OR “Precedex” [Title/Abstract] OR “Medetomidine” [Title/Abstract]

#3. Search (#1 OR #2)

#4. Search “Femoral nerve block” [Title/Abstract]

#5. Search (#3 AND #4)

**Embase**

#1. 'dexmedetomidine'/exp

#2. 'dexmedetomidine':ti,ab OR 'precedex':ti,ab OR 'medetomidine':ti,ab

#3. 'femoral nerve block'/exp

#4. 'femoral nerve block':ti,ab

#5. #1 OR #2

#6. #3 OR #4

#7. #5 AND #6

**Cochrane Library**

#1. MeSH: [dexmedetomidine]

#2. (dexmedetomidine):ti,ab,kw OR (precede):ti,ab,kw OR (medetomidine):ti,ab,kw

#3. #1 OR #2

#4. (femoral nerve block):ti,ab,kw

#5. #3 AND #4

**Web of Science**

#1. TS= (dexmedetomidine OR precedex OR medetomidine)

#2. TS= (femoral nerve block)

#3. #1 AND #2
